# Diversity of Bifidobacteria within the Infant Gut Microbiota

**DOI:** 10.1371/journal.pone.0036957

**Published:** 2012-05-11

**Authors:** Francesca Turroni, Clelia Peano, Daniel A. Pass, Elena Foroni, Marco Severgnini, Marcus J. Claesson, Colm Kerr, Jonathan Hourihane, Deirdre Murray, Fabio Fuligni, Miguel Gueimonde, Abelardo Margolles, Gianluca De Bellis, Paul W. O’Toole, Douwe van Sinderen, Julian R. Marchesi, Marco Ventura

**Affiliations:** 1 Laboratory of Probiogenomics, Department of Genetics, Biology of Microorganisms, Anthropology and Evolution, University of Parma, Parma, Italy; 2 Institute for Biomedical Technologies, National Research Council, Milan, Italy; 3 Cardiff School of Biosciences, Cardiff University, Cardiff, United Kingdom; 4 Alimentary Pharmabiotic Centre and Department of Microbiology, Bioscience Institute, University College Cork, Cork, Ireland; 5 Department of Paediatrics and Child Health, University College Cork, Cork, Ireland; 6 Departamento de Microbiología y Bioquímica de Productos Lacteos, IPLA–CSIC, Villaviciosa, Asturias, Spain; University of Florida, United States of America

## Abstract

**Background:**

The human gastrointestinal tract (GIT) represents one of the most densely populated microbial ecosystems studied to date. Although this microbial consortium has been recognized to have a crucial impact on human health, its precise composition is still subject to intense investigation. Among the GIT microbiota, bifidobacteria represent an important commensal group, being among the first microbial colonizers of the gut. However, the prevalence and diversity of members of the genus *Bifidobacterium* in the infant intestinal microbiota has not yet been fully characterized, while some inconsistencies exist in literature regarding the abundance of this genus.

**Methods/Principal Findings:**

In the current report, we assessed the complexity of the infant intestinal bifidobacterial population by analysis of pyrosequencing data of PCR amplicons derived from two hypervariable regions of the 16 S rRNA gene. Eleven faecal samples were collected from healthy infants of different geographical origins (Italy, Spain or Ireland), feeding type (breast milk or formula) and mode of delivery (vaginal or caesarean delivery), while in four cases, faecal samples of corresponding mothers were also analyzed.

**Conclusions:**

In contrast to several previously published culture-independent studies, our analysis revealed a predominance of bifidobacteria in the infant gut as well as a profile of co-occurrence of bifidobacterial species in the infant’s intestine.

## Introduction

The gastrointestinal microbiota plays a crucial role in health and disease of the host through its impact on nutrition, pathogenesis and immunology [1]. Studies employing murine models have highlighted the critical role played by the gut microbiota in the development of a properly functioning gastrointestinal tract [2], [3]. Furthermore, microbial dysbiosis has been linked to several functional gut disorders, such as inflammatory bowel disease [4], [5], irritable bowel syndrome [6], stomach cancer [7], mucosa-associated lymphoid tissue lymphoma [8], obesity [9], [10] and necrotizing enterocolitis [11]. Recent advances in culture-independent techniques for microbial community analysis have highlighted the diversity, individual variability and complexity of the human gut microbiota [12], [13], [14]. The adult human gut microbiota is considered to be more complex than its infant equivalent, while being stable over time and similar between individuals [12], [15]. In contrast, the infant gut microbiota possesses a relatively simple structure, but is rather unstable over time. There are conflicting reports in the current literature concerning the composition of the gut microbiota of infants [16]–[19]. For a long time bifidobacteria were considered to represent the dominant component of the neonatal gut microbiota, based on both culture-based techniques and analysis using species-specific DNA probes [14], [20]–[24]. However, recent metagenomic studies that investigated the development of the infant gut microbiota revealed low abundance or even apparent absence of bifidobacteria [16], [19]. Mode of delivery as well as type of nutrition, i.e. breast fed vs. bottle fed, are considered to be key factors that provide differential colonization opportunities and thus composition of the neonatal gut microbiota [20], [25]. High levels of bifidobacteria in the infant gut have been associated with the timely and appropriate development and maturation of the immune system [26]. Given the potential for elements of the infant microbiota to impact on this highly complex and dynamic developmental process, there is considerable interest in determining the composition of the infant gut microbiota, including the assessment of the bifidobacterial contribution to such a microbial consortium.

Here, we analysed the microbiota composition of 11 infant subjects from three different geographical areas by pyrosequencing two hypervariable regions of the 16 S rRNA gene. These data provide novel insights into the composition and inter-individual variability of the infant gut microbiota with the identification of bifidobacterial species that likely represent typical infant-associated commensals and that may thus play a role in maintaining the health status of their host.

## Materials and Methods

### Subject Recruitment and Sample Collection

The study was approved by the Clinical Research Ethics Committee of the Cork Teaching Hospitals and by the Ethical Committee of the Regional Asturias Public Health Service (SESPA); informed written consent was obtained from the mothers involved in this study. All subjects were healthy and had not received any antibiotic or probiotic in the previous 3 months. Stool samples consisted of 6–10 gr of fresh faecal material, which was immediately frozen upon collection at −80°C until processed for DNA extraction.

### DNA Extraction and 16 S rDNA Amplification

DNA was extracted according to the protocol described by Apajalahti JH, Särkilahti LK, Mäki BR, Heikkinen JP, Nurminen PH, *et al.* (1998) [27]. Briefly, 1.5 gr of fecal material was suspended in 50 ml of wash buffer (50 mM sodium phosphate buffer [pH 8], 0.1% Tween 80) and mixed for 20 min on a horizontal shaking platform. Fecal debris was removed by centrifugation at 200×g for 15 min. Bacteria in the supernatant were collected by centrifugation at 30,000×g for 15 min at room temperature. Bacterial pellets were suspended in 3 ml of TE buffer (10 mM Tris [pH 8], 1 mM EDTA) and submitted to five freeze-thaw cycles of incubation at −80°C. Recovered bacteria were suspended in 1 ml of lysis buffer (10 mM Tris [pH 8], 5 mM EDTA, 25% [wt/vol] sucrose) and then lysed following incubation at 37°C for one hour in the presence of 200 mg/ml of lysozyme and 25 µg/ml of mutanolysin. Bacterial cell lysis was completed by mechanical treatment placing the bacterial suspension in a tube containing 0.8 g of glass beads (diameter, 106 µm; Sigma). Cells were lysed by shaking the mix on a BioSpec homogenizer at 4°C for 2 min (maximum setting). After the addition of 0.2 ml of 10% [wt/vol] sodium dodecyl sulfate and 20 µl of proteinase K solution (20 mg/ml in TE buffer), the mixture was incubated at 37°C for an additional hour. The cell lysate was extracted twice with an equal volume of chloroform-isoamyl alcohol (24∶1) and then subjected to centrifugation at 6000×g for 10 min at room temperature. DNA was precipitated by the addition of absolute ethanol followed by centrifugation at 10,000×g for 15 min at 4°C. DNA pellet was washed briefly with 70% ethanol, vacuum dried, and dissolved in 1 ml of TE.

Partial 16 S rRNA gene sequences were amplified from extracted DNA using a broad-range, bacterium-specific primer pair Puni (5′-GATGCAACGCGAAGAACC-3′) and P6 (5′-GGTACGGCTACCTTGTTACGA-3′) [28], or employing the bifidobacterial-specific primer pair BIF-specific (5′-GGTGTGAAAGTCCATCGCCT-3′) and Bif seq rev (5′- CTGGACGTGAGGGGCATC-3′) [14]. These primers were designed to include at their 5′ end one of the two adaptor sequences used in the 454-sequencing library preparation protocol (adaptor A and adaptor B) linking a unique TAG barcode of 10 bases to identify different samples (the complete list of the primers used in this study is presented in Table S1). Five different primer pairs for the hyper-variable region V6–V8, bringing five different barcode sequences, were synthesized (Thermo Fisher Scientific, Germany) and used to uniquely distinguish specific amplicon-sample combinations. In this way, it was possible to sequence multiple amplicons pooled in a single picotiter plate lane, though they represented different samples.

The PCR conditions used were 5 min at 95°C, 35 cycles of 30 s at 94°C, 30 s at 55°C and 90 s at 72°C, followed by 10 min at 72°C. Amplification was carried out by using a Verity Thermocycler (Applied Biosystems). The integrity of the PCR amplicons was analyzed by electrophoresis on an Experion workstation (BioRad, UK).

### Pyrosequencing of 16 S rDNA-based Amplicons

The PCR products derived from amplification of specific 16 S rDNA hypervariable regions were purified with Agencourt AMPure XP DNA purification beads (Beckman Coulter Genomics GmbH, Bernried, Germany) in order to remove primer dimers, and then quantified using the Quant-iT PicoGreen dsDNA kit (Invitrogen, Leek, Netherlands).

After the quantification step, amplicons were pooled in equal amounts and fixed to micro beads to be clonally amplified by performing an emulsion PCR following the GS-FLX protocol Titanium emPCR LIB-A (454 LifeSciences, Roche, Branford, CT, USA). Following this amplification step the beads were enriched in order to keep only those carrying identical PCR products on their surface, and then loaded onto a picotiter plate for pyrosequencing reactions according to the GS-FLX Titanium sequencing protocol. The number of sequences produced per amplicon and sample varied significantly due to the fact that we tried to estimate the optimal number of reads to be sequenced in order to capture all possible microbial diversity in the datasets. Thus, the first samples were sequenced in half-plates, while we then moved down to quarters and, finally, to one eighth-plate per sample.

### Sequence-based Microbiota Analysis

Raw sequences obtained from the various amplicon pools were demultiplexed using the sff file utility from 454 Sequencing System Software (version 2.5.3) (454 LifeScience), in order to distinguish between Puni and Bif amplicons derived from the 11 infant samples and the four samples that originated from mothers. In order to allow for a correct multiple alignment for ecology parameter evaluation we selected only the reads starting from the same side of the two amplicons. Specifically, reads starting from the 5′-end of the Bif and the 3′-end of the Puni were selected.

The sff files were processed using QIIME [29] and 454 sequence data were subjected to ‘denoising’ using the associated Denoiser [30] as independent PUNI and BIF datasets. Quality control retained sequences with a length between 350 and 600 bp, mean sequence quality score >25, with truncation of a sequence at the first base if a low quality rolling 50 bp window was found. Presence of homopolymers >6 bp, and sequences with mismatched primers were omitted. The universal 16 S rRNA PUNI dataset contained 193,985 reads (average number of reads per sample: 12,932; range: 4829–47038). The BIF dataset contained 179,414 reads, (average number of reads per sample: 11,960; range 3515–23469). The quantitative data for both Datasets are described in Table S2. In order to calculate downstream diversity measures (alpha and beta diversity indices, Unifrac analysis), PUNI 16 S rRNA OTUs were defined at ≥97% sequence homology and Bif OTUs at ≥99%. The cutoff values were chosen to reflect the broader bacterial-universal amplicon PUNI captured compared to the nuances of the narrow-spectrum of the BIF dataset. All reads were classified to the lowest possible taxonomic rank using QIIME and a reference dataset from the Ribosomal Database Project [31]. Rarefaction curves were obtained by plotting the number of different phylotypes identified against the number of clones sequenced. Co-occurrence analysis was carried out using Ecosim 7 (http://garyentsminger.com/ecosim.htm.) as published by Koening JE, Spor A, Scalfone N, Fricker AD, Stombaugh J. *et al.* (2010) [19].

### Hierarchical Clustering

Operational Taxonomic Units (OTUs) were assigned using uclust [32]. The hierarchical clustering based on population profiles of most common and abundant taxa was done using UPGMA clustering (Unweighted Pair Group Method with Arithmetic mean, also known as average linkage) on the distance matrix of OTU abundance. This resulted in a Newick formatted tree, which was obtained utilizing the QIIME package. Host-bacterial network was constructed as a bipartite graph, in which each node represented either a host sample or a bacterial OTU. Connections were drawn between samples and OTUs, with edge weights defined as the number of sequences from each OTU that occurred in each sample. Networks were visualized using Cytoscape 2.5.2 [33].

### Nucleotide Sequence Accession Numbers

The raw sequences reported in this article have been deposited in the NCBI Short Read Archive (SRA) (SRP007633).

## Results

### Bifidobacteria Dominate Intestinal Microbiota in Infant Subjects

**Figure 1:**
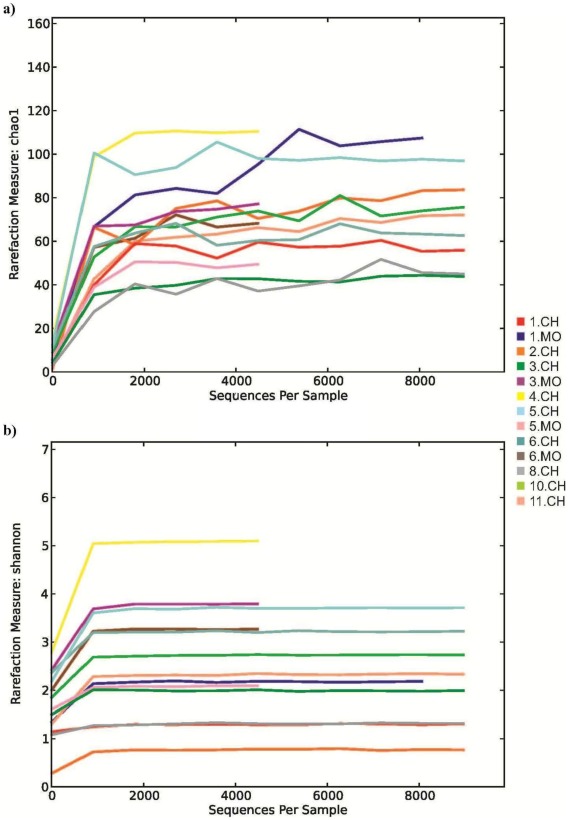
Rarefaction curves generated for 16 S rRNA gene sequences obtained from stool samples of infants and faecal samples of mothers. Panel a represents the rarefaction curves using the Chao index. Panel b displays rarefaction curves using the Shannon index.

**Figure 2:**
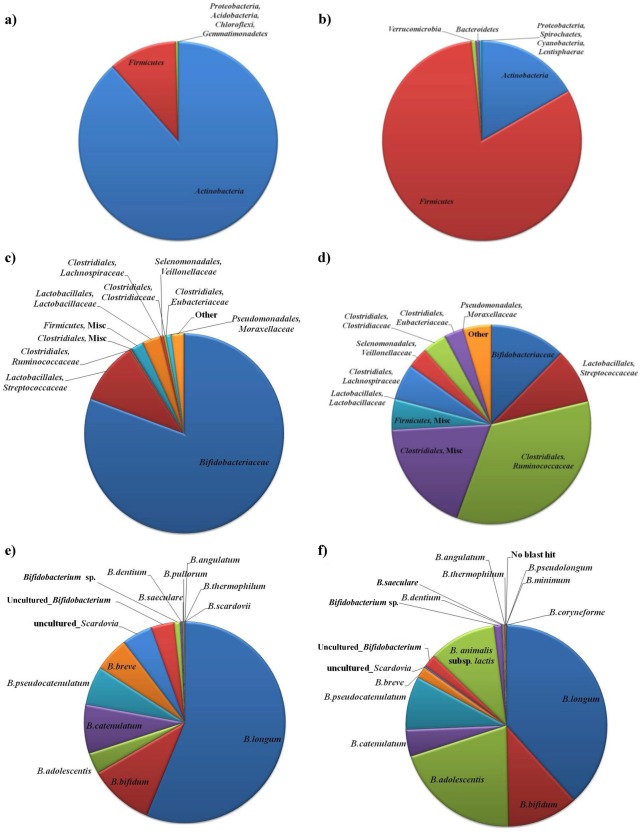
Aggregate microbiota composition at phylum level in faecal samples from infants (panel a), mothers (panel b), at order/family level in infants (panel c) and mothers (panel d), and at *Bifidobacterium* genus level in the same sets of individuals (panels e and f), as indicated. In panels a and b only major taxonomic groups are shown.

Composition of the faecal microbiota retrieved from the cohort (11 samples - three groups of neonatal subjects from Italy, Ireland and Spain; Table 1) was analyzed by sequencing the V6 and V8 regions of the 16 S rRNA genes [34]–[36]. The decrease in the rate of phylotype detection and the plateauing of all the diversity indices for each sample demonstrated that a large part of the diversity in these libraries had been detected (Fig. 1). Notably, the aggregate microbiota obtained for these 11 samples, based on a total of 179,414 reads, shows that the phylum *Actinobacteria* was dominant at 88.5% compared with 11.1% for the phylum *Firmicutes* (Fig. 2a and Table S3). In contrast, when a similar analysis was performed involving fecal samples from the mothers of four of these infants, the *Firmicutes* was the dominant phylum (Fig. 2b), confirming previously published data on the fecal microbiota of adult subjects [13]. Noticeably, the human adult gut microbiota composition was shown to display high variability at inter-subject level, consistent with previously published studies [12]–[13]. Such a high level of microbiota variation between adult subjects might also be responsible for the low levels of detected *Bacteroidetes* in the microbiota of mothers’ fecal samples. This low level of *Bacteroidetes* members in the mother’s fecal samples was confirmed by pyrosequencing of 16 S rDNA amplicons, which had been obtained by employing a previously described primer set [37] (data not shown).

**Table 1:** Sample origin and metadata.

Sample code^1^	Sampling age (Month)	Nutrition	Delivery mode	Nationality
1CH*	3	breast fed	vaginal	Italian
				
4CH	5	breast fed	vaginal	
				
3CH*	3	Both	vaginal	
				
5CH*	3	breast fed	vaginal	
				
2CH	3	breast fed	vaginal	Spanish
				
6CH*	3	breast fed	vaginal	
				
7CH	2	breast fed	c-section	Irish
				
8CH	1.5	bottle fed	vaginal	
				
9CH	2	bottle fed	c-section	
				
10CH	2	bottle fed	vaginal	
				
11CH	2	breast fed	vaginal	
				

1 Infant samples for which a mother sample was analyzed are identified with an asterisk.

The most abundant classes in the infant faecal samples was *Bifidobacteriales*, being present at 80.6%, while second and third most abundant classes were *Lactobacillales* and *Clostridiales* being present at 7.2% and 3.1%, respectively (Fig. 2c).

The genus *Bifidobacterium* currently comprises 37 species [38], [39]. However, the dominant bifidobacterial species detected in the investigated infant faecal samples were *Bifidobacterium longum* and *Bifidobacterium bifidum* at 56.2% and 10.7%, respectively (Fig. 2e). In contrast, analysis of the faecal samples from the mothers showed that *B. longum* and *B. adolescentis* were the dominant species, constituting 38.2% and 20.3%, respectively, of the sequences that were assigned to the genus *Bifidobacterium* (Fig. 2f).

### Interindividual Variability Detected in the Infant Gut Microbiota

In order to investigate if and to what extent bacterial communities were different between individuals, the significance test in UniFrac [40] was applied and raw P-values determined (P-values≤0.05 were considered to be statistical significant). This method was used to evaluate if the cluster distribution of the sequences in the different faecal samples differs from random expectations. Principal Coordinate Analysis (PCoA), applied using the UniFrac program, showed that axis 1 (PCo1) explained 20.6% of the variability, while PCoA axis 2 explained 14.03% (PCo2).

**Figure 3:**
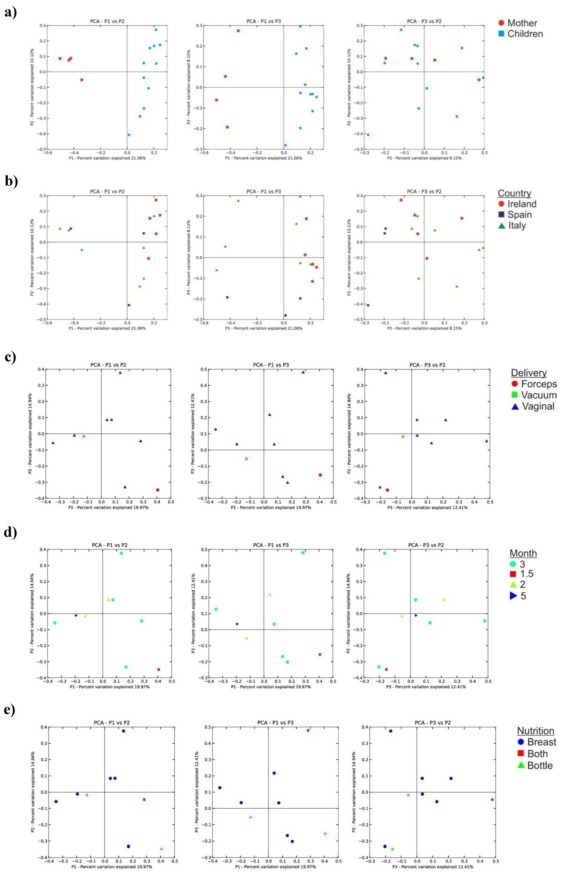
Principal Coordinate Analysis (PCoA) based on the phylotypes identified from different subjects (panel a), different geographical regions (panel b) different mode of delivery (panel c), different age (panel d), and different feeding type (panel e). Percentages shown along the axes represent the proportion of dissimilarities captured by the axes. Each circle represents the 16 S rRNA gene sequences from each sample, which have different colour and shape according to the subject and the age (infant vs. mother), respectively.

The PCo1 shows positive values for all infant samples and negative values for all mother samples, which is associated with the bifidobacterial prevalence in the infant gut microbiota as opposed to the situation in the samples obtained from the mothers (Fig. 3a). The PCo2 shows positive or negative values depending on geographical origin (Fig. 3b); age (Fig. 3d); feeding type (Fig. 3e) allowing an obvious clustering of infant samples. This component elucidates an interesting correlation between age, geographical origin and feeding on one hand and the infant gut microbiome composition on the other. However, the low number of individuals included in this study limits a broader extension of the interpretation of these data.

Finally, the PCo3 shows the inter-individual variability among samples both for mothers and infants; from the plots it is evident that the values are dispersed along the x and y axis without any correlation between any of the classification parameters considered.

In addition, the raw P values generated using the Unifrac significance test were all less than 0.001 and further re-enforce the view that these are significantly different communities.

**Figure 4:**
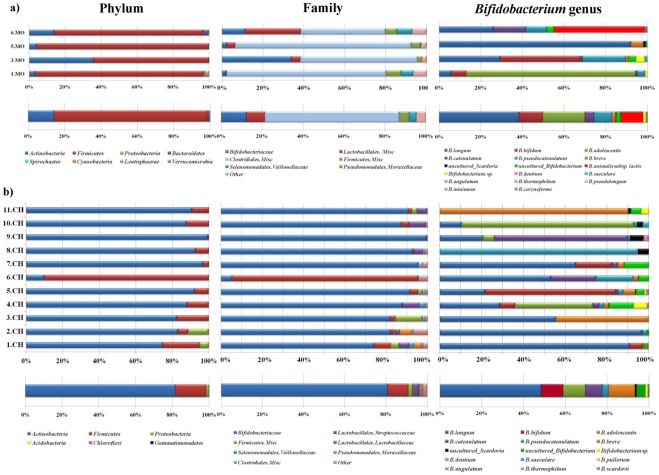
Inter-individual variation in infant stool samples in the proportion of the major microbial phylum/family/species. The core faecal microbiota of infant subjects at the levels of phylum, family and genus is indicated at the bottom of each inset.

The individual composition of infant gut datasets revealed a large conservation of members of the *Actinobacteria* with a high proportion belonging to the family *Bifidobacteriaceae* ranging from 74% to 99.3%, except for one sample where the proportion was just 5.3% (Fig. 4). Despite this high abundance of phylotypes that belong to the genus *Bifidobacterium*, a significant species variation is noticeable. In fact, whereas the aggregate microbiota was dominated by *B. longum* species, the individual composition datasets showed extraordinary variation, with the proportion of *B. longum* ranging from 21.7% to 90.6%. The proportion of five other major bifidobacterial taxa varied substantially between individuals. For example, in these 11 gut communities, the *B. breve* species was always detected in a range between 0.3% and 44.4% of total reads, with an average of 5.5% (Fig. 4). In contrast, other bifidobacterial taxa such as *B. adolescentis* was detected in a relatively high average percentage (3.4%), but was only present in a small number of the subjects (two out 11, Fig. 4). Notably, 3.7% of the total number of reads were assigned to bifidobacteria that are very closely related (≥99% sequence identity) to uncultured bifidobacterial phylotypes retrieved from human faecal samples (NCBI source). In addition, a small number of reads, representing 0.23% of the total number of bifidobacterial sequences had not been identified previously. These novel phylotypes are closely related to the *B. adolescentis* phylogenetic group. These sequences fall into two different operational taxonomic units, which based on the criteria described above, would constitute novel bifidobacterial phylotypes.

We employed network-based analyses to map gut microbial community composition and structure onto geographical origin, mode of delivery, type of feeding and age, thereby complementing the PCoA analyses. Network analyses indicated a high number of shared OTUs between individuals highlighting a co-occurring community (Fig. S1). Taxonomic investigation presented a population distinctly dominated by *Bifidobacterium longum* albeit in varying proportion. Other consistently occurring taxa were *B. bifidium*, *B. breve* and *B. psuedocatenulum* although the variance in their presence may be influenced by factors currently unidentified.

### Similarities and Differences Among Population Patterns

**Figure 5:**
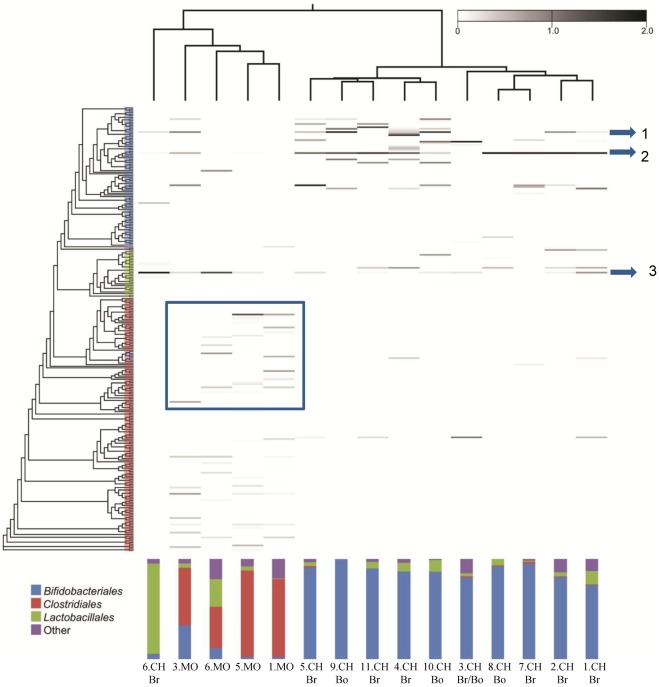
Number of sequences per phylotype for each sample. The y axis is a neighbour-joining phylogenetic tree containing one representative of each of the OTUs detected in this study; each row represents a different OTU. Increasing darkness of the grayscale corresponds to higher estimated relative abundance. The most conserved OTUs between samples are marked with an arrow. Arrow 1 represents the OTUs corresponding to *B. longum*, arrow 2 indicates the OTUs of *B. pseudocatenulatum*, arrow 3 highlights the phylotypes belonging to *Streptococcus thermophilus*. The boxed area includes those phylotypes belonging to *Ruminococcaceae* and *Clostridiales* taxa. The feeding method is indicated for each subject; Br, breast-fed; Bo, bottle fed.

We analyzed the similarities and differences in the composition of all 11 samples by hierarchical clustering based on population profiles of the most common and abundant taxa (Fig. 5). The clustering patterns are also reflected in the corresponding dendrogram (Fig. 5), highlighting several interesting features of the colonization program, and revealing a close relationship between the faecal microbiota composition of breast fed infants and that of bottle fed infants, suggesting that bifidobacterial infant gut colonization may not be influenced by diet alone. Furthermore, samples from identical geographical origins (i.e., Italy, Ireland or Spain) display dissimilar microbial profiles (Fig. 5), suggesting that bifidobacterial infant gut colonization is not influenced by the environment to which neonates of different geographical areas are exposed. Surprisingly, the gut microbiota of one of the infant samples (CH6) was shown to be very dissimilar from that obtained from the other infant samples. It presents a high abundance of OTUs corresponding to lactobacillae. Further analysis also revealed a rather distant relationship between the gut microbiota of the mother and her child, in fact apart for the pair CH6 and MO6 the other mother-child pairs do not branch together (Fig. 5).

Notably, the heat-map represented in Figure 5 displays the existence of common OTUs between infant- and adult-samples, including OTUs corresponding to *B. longum* and *B. pseudocatenulatum* species as evidenced by the arrows on the right side of the figure. Otherwise specific OTUs including *Ruminococcaceae* and uncultured *Clostridiales* appear to be specifically present in the adult subjects, as highlighted by the bands included in the box (Fig. 5).

### Intersection of the Gut Microbiota of Mothers vs. Infants

It has been postulated that bacterial transmission may take place from the mother to the newborn/infant through direct contact with maternal microbiota during birth and through breast milk during lactation [16], [19], [41]. In order to explore if a similar scenario may be applicable to the bifidobacterial population, we analyzed the distribution of bifidobacterial phylotypes using PCoA. In this analysis, the bifidobacterial communities from each of the four mother samples were found to be inter-dispersed in the same quadrants of the plot of the bifidobacterial population of the 11 infant samples, which supports the notion that part of the identified variability in the bifidobacterial population between these sets of samples might be explained by inter-participant differences (Fig. 3a). Common OTUs corresponding to *B. longum*, *B. bifidum* and *B. breve* species were identified to be commonly distributed between all four mother-infant pairs analyzed, whereas *B. dentium*, *B. catenulatum* taxa were shown to be shared between only a single mother-infant pair (Fig. 4).

### Species Co-occurrence and Exclusions

**Figure 6:**
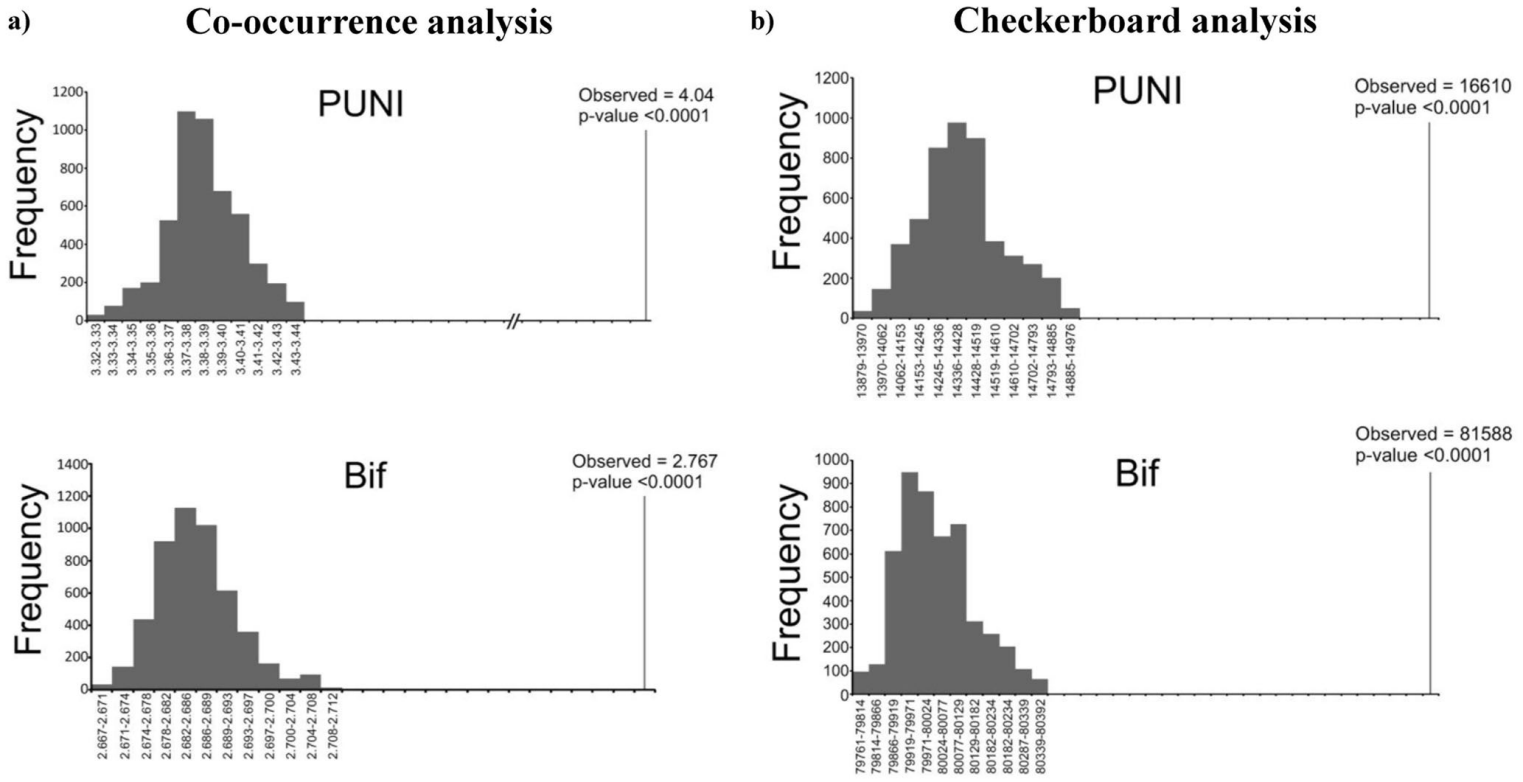
Analysis of species co-occurrence/exclusion. Panel a represents the C-score distribution for observed and randomized OTU occurrence in each sample. Panel b shows the checkerboard indices for observed and randomized OTU occurrence. Values for the observed distributions are indicated on the X axis.

Initial OTU-based cluster analysis indicated the existence of co-occurrence of bifidobacterial species, and we therefore investigated if the infant microbiota follows community assembly rules. In order to evaluate OTU coexistence we evaluated both the C-score, indicating the trend of one species to exclude another from the same ecological niche [42], as well as checkerboard measures, which represent the number of species pairs that never co-occur [43]. In order to evaluate the significance of the scores achieved from the dataset, we compared the C-score and the checkerboard indices from actual data with scores obtained from 5000 communities assembled randomly from the same OTU data. The obtained results showed that the C-score for the real dataset was 2.767, which is higher than that achieved using randomized data (p-value <10^−4^). Furthermore, the checkerboard measure for the microbial communities (16610) was higher than the checkerboard measures obtained for randomized data (13879–14976) (Fig. 6). Although these data suggests that the neonatal gut microbiota is composed of interacting microbial consortia, rather than randomly assembled bacterial populations, the number of samples analysed is too low in order to draw a conclusive and decisive assertion.

### Analysis of the Core Microbiota of Infant Subjects

We identified and compared the core microbiota of infant subjects with that of adults. Adult samples were represented by faecal material obtained from a subset of four mothers, which was collected at the same time as the samples from infants. The gut microbiota composition of infant subjects appears to be mainly linked to the diet rather than to the age of the subjects. Despite the age variation of the infant subjects employed in this study (Table 1), all individuals were still fed on a mainly milk-based diet.

We defined the core bifidobacterial microbiota as the composite of the unique, bifidobacteria-assigned sequences present in at least one-half of the subjects following the procedure described by Tap J, Mondot S, Levenez F, Pelletier E, Caron C, *et al*. (2009) [15]. Taxonomical classification of the two datasets at phylum, genus (i.e.: *Bifidobacterium*) levels or focused on bifidobacterial species revealed large differences between infants and adults. Notably, more than half of the infant core microbiota that corresponded to *Actinobacteria* was formed by the genus *Bifidobacterium* and the species *B. breve* and *B. bifidum*, compared to less than 1% in the adult subjects (Figs. 2 and 4). With respect to the genus *Bifidobacterium*, no bifidobacterial sequences were uniquely identified in the faecal samples of infants or adults. These findings reinforce the hypothesis of a transmission of elements of the mother’s bifidobacterial population to the infant through faecal contamination and/or milk feeding [44]–[45].

## Discussion

This study represents one of the most exhaustive sampling of the composition of the infant gut with particular focus on the bifidobacterial population reported to date. Various publications have reported that the infant/human gut contains at best low numbers of bifidobacteria, which is in dramatic contrast with findings from both culture-dependent approaches [14], [20], [32] and some culture-independent approaches [46]–[47]. In the current report we show that the paucity of bifidobacterial species described by culture-independent investigations is most likely due to technical biases, in particular those related to DNA extraction protocols and/or the PCR primers used. Therefore, caution must be applied in the interpretation of the results obtained by various published metagenomic studies of the microbial biodiversity of the infant gut [16]. Nevertheless, even if the DNA extraction and PCR amplification protocols, as presented in this study, demonstrated good potential for bifidobacterial profiling of human gut ecosystems, these still come with some limitations, such as the potential effects on amplification yields due to increased sized amplicons [24], [38]. Also, it is possible that the set of primers described here display a reduced efficiency of amplification of 16 S rRNA genes of other component of the human gut microbiota. However, it is important to highlight that it will be near to impossible to design a PCR primer set that is able to generate an equally efficient amplification yield of 16 S rRNA gene sequences across all components of the human gut microbiota [48].

This study reinforces the notion of bifidobacteria as a predominant component of the infant gut microbiota, as determined from the analysis of infant stool samples, thereby implicating this bacterial group as one of the main microbial candidates to affect the physiology/immunology of their infant host. The gut microbiota of these infant samples shows a substantially different composition to that of adult subjects, particularly because of a high abundance of bifidobacteria and a lower proportion of *Firmicutes*.

Noticeably, the C-score and checkerboard analyses strongly support a non-random pattern of community assembly. It is already known for quite some time that the intestinal gut microbiota is composed of syntrophic as well as antagonistic members [49]. Thus, it is likely that such ecological relationships explain the non-random associations of species constituting the infant gut bifidobacterial population. The particular co-existence of bifidobacterial taxa might represent a fascinating example of co-evolution by bacteria-host and diet. Previous genomic analyses have described how different bifidobacterial species (e.g., *B. bifidum* and *B. longum* subsp. *infantis*) are genetically adapted to utilize host-produced glycans like mucins and human milk oligosaccharides [50]–[53]. In such an environment it may be envisaged that such species establish interesting nutrient-based co-operations, where a microbial species liberates host-glycan components that are then internalized and metabolized by another bacterial species.

Nevertheless, due to the fact that only 11 fecal samples were analyzed coupled to the observed high level of microbiota variation and the different geographical origin of the subjects involved in this study, we should apply caution in drawing conclusions as regards to the general infant gut microbiome. In this context, these limitations may influence the results achieved by ecological analyses such as the determination of a core microbiome, or the effect of diet (e.g., breast fed vs. bottle fed) and the interpretation of the inter-individual variability of the gut microbiota.

The immediate future perspective of this work will be the infant gut metagenomic analysis to identify the existence of particular gene repertoires and gene networks assisting the co-operation of synergic bifidobacterial species in the colonization and metabolism of infant diet components as well as host-derived nutrients, such as human milk.

In contrast to previous publications on infant faecal microbiota profiling efforts, this study clearly implicates bifidobacteria in shaping and influencing the gut environment at this early stage of life. Understanding the parameters that influence colonization, development and composition of the microbiota from a very early stage following birth, may be crucial for the development of strategies that guide formation of health-sustaining or -promoting microbiota to elicit its beneficial activities into subsequent life stages.

## Supporting Information

Figure S1 **Simplified cartoon illustration of possible host-gut microbe networks.** Network diagrams are colour-coded by geographical origin (panel A), type of feeding (panel B), type of delivery (panel C) and age of the subjects (panel D).
(TIFF)

Table S1 **Primers used in this study.**
(DOC)

Table S2 **Quantitative data of the 16 S rRNA PUNI and BIF datasets.**
(DOC)

Table S3 **Relative amounts of the most dominant taxa between the samples analysed.**
(DOC)
