# Supplementary figures and images for: Diversity of Bifidobacteria within the Infant Gut Microbiota
Source: PLoS One. 2012 May 11;7(5):e36957. doi: 10.1371/journal.pone.0036957 (PMC3350489; doi:10.1371/journal.pone.0036957)

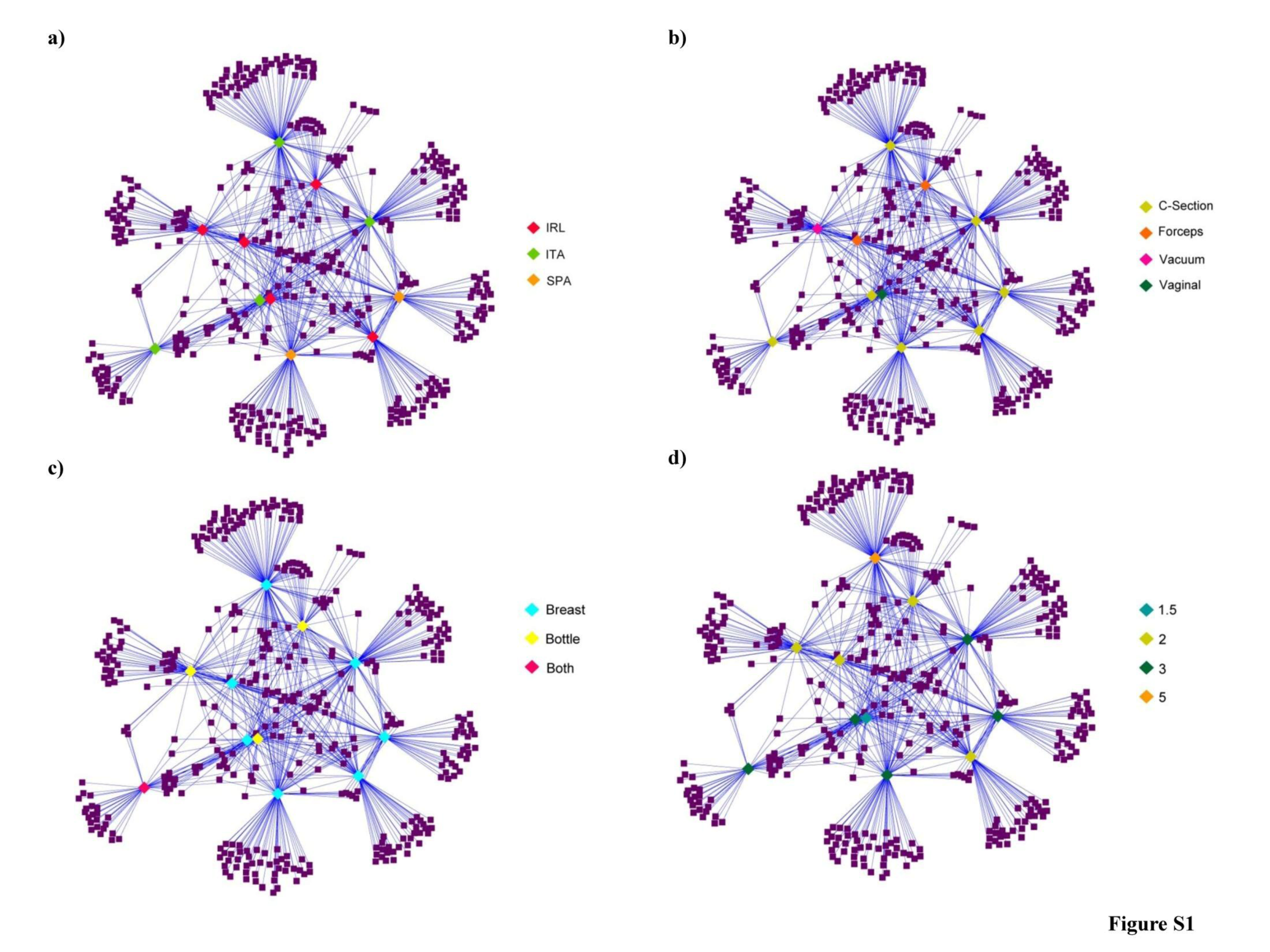

Supplement: Figure S1 — Simplified cartoon illustration of possible host-gut microbe networks. Network diagrams are colour-coded by geographical origin (panel A), type of feeding (panel B), type of delivery (panel C) and age of the subjects (panel D). (TIFF) [file pone.0036957.s001.tif]
