# Supplementary material for: Diversity of Bifidobacteria within the Infant Gut Microbiota
Source: PLoS One. 2012 May 11;7(5):e36957. doi: 10.1371/journal.pone.0036957 (PMC3350489; doi:10.1371/journal.pone.0036957)
Supplement: Table S1 — Primers used in this study. (DOC) [file pone.0036957.s002.doc]

**Table S1.** Primers used in this study

| Primer name | 454 Primer | | MID | MID tag | specific primer (5' -> 3') | Full Sequence (5' -> 3') |
| --- | --- | --- | --- | --- | --- | --- |
| Bif_Spec | A | CGTATCGCCTCCCTCGCGCCATCAG | Mid1 | ACGAGTGCGT | GGTGTGAAAGTCCATCGCCT | CGTATCGCCTCCCTCGCGCCATCAGACGAGTGCGTGGTGTGAAAGTCCATCGCCT |
| Bif_Spec | A | CGTATCGCCTCCCTCGCGCCATCAG | Mid2 | ACGCTCGACA | GGTGTGAAAGTCCATCGCCT | CGTATCGCCTCCCTCGCGCCATCAGACGCTCGACAGGTGTGAAAGTCCATCGCCT |
| Bif_Spec | A | CGTATCGCCTCCCTCGCGCCATCAG | Mid3 | AGACGCACTC | GGTGTGAAAGTCCATCGCCT | CGTATCGCCTCCCTCGCGCCATCAGAGACGCACTCGGTGTGAAAGTCCATCGCCT |
| Bif_Spec | A | CGTATCGCCTCCCTCGCGCCATCAG | Mid4 | AGCACTGTAG | GGTGTGAAAGTCCATCGCCT | CGTATCGCCTCCCTCGCGCCATCAGAGCACTGTAGGGTGTGAAAGTCCATCGCCT |
| Bif_Spec | A | CGTATCGCCTCCCTCGCGCCATCAG | Mid5 | ATCAGACACG | GGTGTGAAAGTCCATCGCCT | CGTATCGCCTCCCTCGCGCCATCAGATCAGACACGGGTGTGAAAGTCCATCGCCT |
|  |  |  |  |  |  |  |
| Bif_Seq_rev | B | CTATGCGCCTTGCCAGCCCGCTCAG | Mid1 | ACGAGTGCGT | CTGGACGTGAGGGGCATG | CTATGCGCCTTGCCAGCCCGCTCAGACGAGTGCGTCTGGACGTGAGGGGCATG |
| Bif_Seq_rev | B | CTATGCGCCTTGCCAGCCCGCTCAG | Mid2 | ACGCTCGACA | CTGGACGTGAGGGGCATG | CTATGCGCCTTGCCAGCCCGCTCAGACGCTCGACACTGGACGTGAGGGGCATG |
| Bif_Seq_rev | B | CTATGCGCCTTGCCAGCCCGCTCAG | Mid3 | AGACGCACTC | CTGGACGTGAGGGGCATG | CTATGCGCCTTGCCAGCCCGCTCAGAGACGCACTCCTGGACGTGAGGGGCATG |
| Bif_Seq_rev | B | CTATGCGCCTTGCCAGCCCGCTCAG | Mid4 | AGCACTGTAG | CTGGACGTGAGGGGCATG | CTATGCGCCTTGCCAGCCCGCTCAGAGCACTGTAGCTGGACGTGAGGGGCATG |
| Bif_Seq_rev | B | CTATGCGCCTTGCCAGCCCGCTCAG | Mid5 | ATCAGACACG | CTGGACGTGAGGGGCATG | CTATGCGCCTTGCCAGCCCGCTCAGATCAGACACGCTGGACGTGAGGGGCATG |
|  |  |  |  |  |  |  |
| Puni | A | CGTATCGCCTCCCTCGCGCCATCAG | Mid1 | ACGAGTGCGT | GATGCAACGCGAAGAACC | CGTATCGCCTCCCTCGCGCCATCAGACGAGTGCGTGATGCAACGCGAAGAACC |
| Puni | A | CGTATCGCCTCCCTCGCGCCATCAG | Mid2 | ACGCTCGACA | GATGCAACGCGAAGAACC | CGTATCGCCTCCCTCGCGCCATCAGACGCTCGACAGATGCAACGCGAAGAACC |
| Puni | A | CGTATCGCCTCCCTCGCGCCATCAG | Mid3 | AGACGCACTC | GATGCAACGCGAAGAACC | CGTATCGCCTCCCTCGCGCCATCAGAGACGCACTCGATGCAACGCGAAGAACC |
| Puni | A | CGTATCGCCTCCCTCGCGCCATCAG | Mid4 | AGCACTGTAG | GATGCAACGCGAAGAACC | CGTATCGCCTCCCTCGCGCCATCAGAGCACTGTAGGATGCAACGCGAAGAACC |
| Puni | A | CGTATCGCCTCCCTCGCGCCATCAG | Mid5 | ATCAGACACG | GATGCAACGCGAAGAACC | CGTATCGCCTCCCTCGCGCCATCAGATCAGACACGGATGCAACGCGAAGAACC |
|  |  |  |  |  |  |  |
| P6 | B | CTATGCGCCTTGCCAGCCCGCTCAG | Mid1 | ACGAGTGCGT | GTACGGCTACCTTGTTACGA | CTATGCGCCTTGCCAGCCCGCTCAGACGAGTGCGTGTACGGCTACCTTGTTACGA |
| P6 | B | CTATGCGCCTTGCCAGCCCGCTCAG | Mid2 | ACGCTCGACA | GTACGGCTACCTTGTTACGA | CTATGCGCCTTGCCAGCCCGCTCAGACGCTCGACAGTACGGCTACCTTGTTACGA |
| P6 | B | CTATGCGCCTTGCCAGCCCGCTCAG | Mid3 | AGACGCACTC | GTACGGCTACCTTGTTACGA | CTATGCGCCTTGCCAGCCCGCTCAGAGACGCACTCGTACGGCTACCTTGTTACGA |
| P6 | B | CTATGCGCCTTGCCAGCCCGCTCAG | Mid4 | AGCACTGTAG | GTACGGCTACCTTGTTACGA | CTATGCGCCTTGCCAGCCCGCTCAGAGCACTGTAGGTACGGCTACCTTGTTACGA |
| P6 | B | CTATGCGCCTTGCCAGCCCGCTCAG | Mid5 | ATCAGACACG | GTACGGCTACCTTGTTACGA | CTATGCGCCTTGCCAGCCCGCTCAGATCAGACACGGTACGGCTACCTTGTTACGA |
