# Supplementary material for: Diversity of Bifidobacteria within the Infant Gut Microbiota
Source: PLoS One. 2012 May 11;7(5):e36957. doi: 10.1371/journal.pone.0036957 (PMC3350489; doi:10.1371/journal.pone.0036957)
Supplement: Table S2 — Quantitative data of the 16 S rRNA PUNI and BIF datasets. (DOC) [file pone.0036957.s003.doc]

**Table S2**. Quantitative data of the 16S rRNA PUNI and BIF datasets

| Dataset | Sample | Number of reads | Number of reads removed because of: | | | | | | Final read number | Reduced by (%) |
| --- | --- | --- | --- | --- | --- | --- | --- | --- | --- | --- |
|  |  |  | Outside bounds (350-600) | Ambiguous bases | Mean quality <25 | Homopolymer runs >6bp | Primer mismatch | Low quality window truncation results in <350bp |  |  |
| **BIF** | 2CH | 98463 | 32861 | 9219 | 101 | 47 | 384 | 27312 | 47042 | 52.22 |
|  | 1MO | 19881 |  |  |  |  |  |  | 8404 | 57.73 |
|  | 3MO | 11978 |  |  |  |  |  |  | 4947 | 58.70 |
|  | 8CH | 50702 | 34427 | 12306 | 74 | 49 | 689 | 40810 | 25262 | 50.18 |
|  | 3CH | 78204 |  |  |  |  |  |  | 20899 | 73.28 |
|  | 1CH | 15554 |  |  |  |  |  |  | 9944 | 36.07 |
|  | 10CH | 33190 | 15361 | 6812 | 32 | 19 | 568 | 13523 | 11721 | 64.69 |
|  | 6MO | 15173 |  |  |  |  |  |  | 5286 | 65.16 |
|  | 7CH | 5906 |  |  |  |  |  |  | 945 | 84.00 |
|  | 6CH | 28284 | 8396 | 5240 | 51 | 12 | 474 | 9409 | 10267 | 63.70 |
|  | 5MO | 10401 |  |  |  |  |  |  | 4830 | 53.56 |
|  | 4CH | 38243 | 20691 | 4249 | 15 | 8 | 0 | 12487 | 4953 | 87.05 |
|  | 9CH | 6353 |  |  |  |  |  |  | 2193 | 65.48 |
|  | 11CH | 37744 | 24373 | 8903 | 39 | 23 | 603 | 21560 | 13767 | 63.53 |
|  | 5CH | 40484 |  |  |  |  |  |  | 8954 | 77.88 |
|  | **TOTAL** | **490560** | **136109** | **46729** | **312** | **158** | **3102** | **125101** | **179414** | **63.43** |
| **PUNI** | 2CH | 4361 | 29399 | 6717 | 75 | 114 | 0 | 23912 | 2259 | 48.20 |
|  | 1MO | 56313 |  |  |  |  |  |  | 16398 | 70.88 |
|  | 3MO | 36045 |  |  |  |  |  |  | 17845 | 50.49 |
|  | 8CH | 8992 | 39533 | 6302 | 123 | 57 | 51 | 50661 | 2470 | 72.53 |
|  | 3CH | 96387 |  |  |  |  |  |  | 23475 | 75.65 |
|  | 1CH | 38352 |  |  |  |  |  |  | 21059 | 45.09 |
|  | 10CH | 28096 | 22941 | 5564 | 21 | 109 | 286 | 27062 | 14719 | 47.61 |
|  | 6MO | 24504 |  |  |  |  |  |  | 11366 | 53.62 |
|  | 7CH | 38244 |  |  |  |  |  |  | 8774 | 77.06 |
|  | 6CH | 31529 | 18156 | 8333 | 49 | 93 | 193 | 12901 | 11638 | 63.09 |
|  | 5MO | 26912 |  |  |  |  |  |  | 7072 | 73.72 |
|  | 4CH | 24666 | 15851 | 2329 | 14 | 99 | 0 | 15290 | 15776 | 36.04 |
|  | 9CH | 28208 |  |  |  |  |  |  | 3515 | 87.54 |
|  | 11CH | 36897 | 19156 | 4656 | 11 | 90 | 457 | 25366 | 20526 | 44.37 |
|  | 5CH | 50461 |  |  |  |  |  |  | 17093 | 66.13 |
|  | **TOTAL** | **525606** | **145036** | **33901** | **293** | **562** | **987** | **155192** | **191726** | **63.5229** |
