# Supplementary material for: Diversity of Bifidobacteria within the Infant Gut Microbiota
Source: PLoS One. 2012 May 11;7(5):e36957. doi: 10.1371/journal.pone.0036957 (PMC3350489; doi:10.1371/journal.pone.0036957)
Supplement: Table S3 — Relative amounts of the most dominant taxa between the samples analysed. (DOC) [file pone.0036957.s004.doc]

**Table S3**. Relative amounts of the most dominant taxa between the samples analysed.

| **Samples/Taxa** | **1CH** | **2CH** | **3CH** | **4CH** | **5CH** | **6CH** | **7CH** | **8CH** | **9CH** | **10CH** | **11CH** | **1MO** | **3MO** | **5MO** | **6MO** | **Total** |
| --- | --- | --- | --- | --- | --- | --- | --- | --- | --- | --- | --- | --- | --- | --- | --- | --- |
| *Actinobacteria* | 15716 | 1875 | 19350 | 3094 | 15772 | 1128 | 8484 | 2289 | 15686 | 12898 | 18607 | 570 | 6369 | 287 | 1571 | 123696 |
| *Firmicutes* | 4292 | 127 | 4074 | 417 | 1302 | 10497 | 285 | 181 | 78 | 1815 | 1902 | 15300 | 11463 | 6766 | 9401 | 67900 |
| *proteobacteria* | 1051 | 249 | 45 | 4 | 19 | 11 | 5 | 0 | 12 | 5 | 17 | 80 | 12 | 9 | 37 | 1556 |
| *Verrucomicrobia* | 0 | 0 | 0 | 0 | 0 | 0 | 0 | 0 | 0 | 0 | 0 | 381 | 0 | 0 | 1 | 382 |
| *Bacteroidetes* | 0 | 0 | 0 | 0 | 0 | 0 | 0 | 0 | 0 | 0 | 0 | 9 | 1 | 8 | 320 | 338 |
| *Spirochaetes* | 0 | 0 | 0 | 0 | 0 | 0 | 0 | 0 | 0 | 0 | 0 | 57 | 0 | 2 | 32 | 91 |
| *Acidobacteria* | 0 | 5 | 0 | 0 | 0 | 0 | 0 | 0 | 0 | 0 | 0 | 0 | 0 | 0 | 0 | 5 |
| *Chloroflexi* | 0 | 2 | 0 | 0 | 0 | 0 | 0 | 0 | 0 | 0 | 0 | 0 | 0 | 0 | 0 | 2 |
| *Cyanobacteria* | 0 | 0 | 0 | 0 | 0 | 0 | 0 | 0 | 0 | 0 | 0 | 0 | 0 | 0 | 2 | 2 |
| *Gemmatimonadetes* | 0 | 1 | 0 | 0 | 0 | 0 | 0 | 0 | 0 | 0 | 0 | 0 | 0 | 0 | 0 | 1 |
| *Lentisphaerae* | 0 | 0 | 0 | 0 | 0 | 0 | 0 | 0 | 0 | 0 | 0 | 1 | 0 | 0 | 0 | 1 |
| Total no | 21059 | 2259 | 23469 | 3515 | 17093 | 11636 | 8774 | 2470 | 15776 | 14718 | 20526 | 16398 | 17845 | 7072 | 11364 | 193974 |
